# Spatial transcriptomic analysis of neurons at the glioma infiltration front: A case study

**DOI:** 10.1111/bpa.70154

**Published:** 2026-09-29

**Authors:** Kaishi Satomi, Hirokazu Takami, Hiromi Onizuka, Kuniaki Saito, Takako Yoshioka, Motoo Nagane, Yutaka Suzuki, Koichi Ichimura, Junji Shibahara

**Affiliations:** ^1^ Department of Pathology Kyorin University School of Medicine Tokyo Japan; ^2^ Department of Neurosurgery, Faculty of Medicine The University of Tokyo Tokyo Japan; ^3^ Department of Neurosurgery Kyorin University School of Medicine Tokyo Japan; ^4^ Department of Pathology National Center for Child Health and Development Tokyo Japan; ^5^ Department of Computational Biology and Medical Science, Graduate School of Frontier Science The University of Tokyo Tokyo Japan

**Keywords:** glioblastoma, IDH‐wildtype, IGFBP5, spatial transcriptomics, TGFβ, Xenium In Situ

## Abstract

Spatial transcriptomics reveals localized IGFBP5 expression and TGFβ‐related signaling in neurons within glioma‐infiltrated tissue.

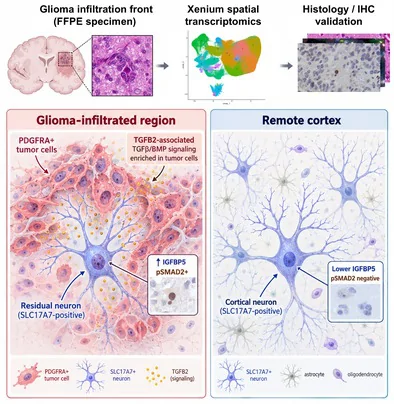

Recent studies have demonstrated that functional neuron–glioma interactions contribute to glioma progression through synapse‐like communication [1, 2, 3]. However, previous studies have primarily examined the effects of neurons on tumor cells, whereas the effects of tumor cells on surrounding neurons remain incompletely understood.

Spatial transcriptomics enables simultaneous evaluation of morphology and gene expression within preserved tissue architecture in human tumors [4]. The Xenium In Situ platform (10x Genomics, Pleasanton, CA, USA) enables visualization of transcript localization at near‐cellular resolution while preserving histopathological context, although important platform‐dependent analytical limitations remain [5].

Formalin‐fixed paraffin‐embedded tissue from a surgically resected tumor in a Japanese man in his 80s at Kyorin University Hospital was analyzed using the Xenium In Situ platform (10x Genomics) with the Human Brain Gene Expression Panel comprising 366 genes. The tumor was diagnosed as glioblastoma, IDH‐wildtype, based on the integrated histopathological and molecular findings according to the WHO Classification of Central Nervous System Tumors, 5th edition. DNA methylation profiling using the Epignostix CNS Tumor Classifier version 12.8.1 (Epignostix GmbH, Heidelberg, Germany) yielded a calibrated score of 0.88478 for “pediatric‐type diffuse high‐grade gliomas” but did not result in assignment to a specific methylation class. Therefore, we regarded this methylation result as supportive molecular information rather than sufficient evidence to reclassify the tumor as a specific pediatric‐type diffuse high‐grade glioma (Figure S1A). Copy number analysis further demonstrated *PDGFRA* amplification and homozygous deletion of *CDKN2A* and *CDKN2B* (Figure S1B). Data were analyzed using Xenium Explorer version 4.1.1 (10x Genomics), R version 4.3.3 (R Foundation for Statistical Computing, Vienna, Austria), utilizing Seurat version 5.1.0, and pathway enrichment workflows. To further evaluate the pSMAD2 findings, four additional adult high‐grade diffuse glioma cases with glioma‐infiltrated and remote cortical regions were examined using immunohistochemistry. pSMAD2‐positive neurons were quantified in 10 fields per region and compared using a two‐sided Mann–Whitney *U* test. The study was approved by the institutional ethics committee (R05‐188).

Unsupervised clustering identified 18 clusters representing tumor cells, neurons, astrocytes, oligodendrocytes, endothelial cells, and immune‐associated cell populations (Figure 1A). The inferred cellular distributions were morphologically consistent with the infiltrative histological architecture of the tissue sections (Figure 1B). Tumor cell clusters demonstrated high *PDGFRA* expression (Figure S2), consistent with *PDGFRA* amplification. Spatial transcriptomic visualization demonstrated distinct spatial localization patterns between Clusters 0 and 1 tumor cell populations, indicating spatial intratumoral transcriptional heterogeneity among *PDGFRA*‐expressing tumor cells (Figure 1C,D). *TGFB2* was expressed in several tumor‐associated clusters, whereas *POSTN* was enriched in a distinct tumor cell subset corresponding predominantly to Cluster 1 (Figure S3). Spatial transcriptomic visualization further demonstrated that POSTN‐expressing Cluster 1 tumor cells showed a distinct spatial distribution, with preferential localization in relatively deep tumor regions, including areas containing vascular structures (Figures 1E and S3).

**FIGURE 1:**
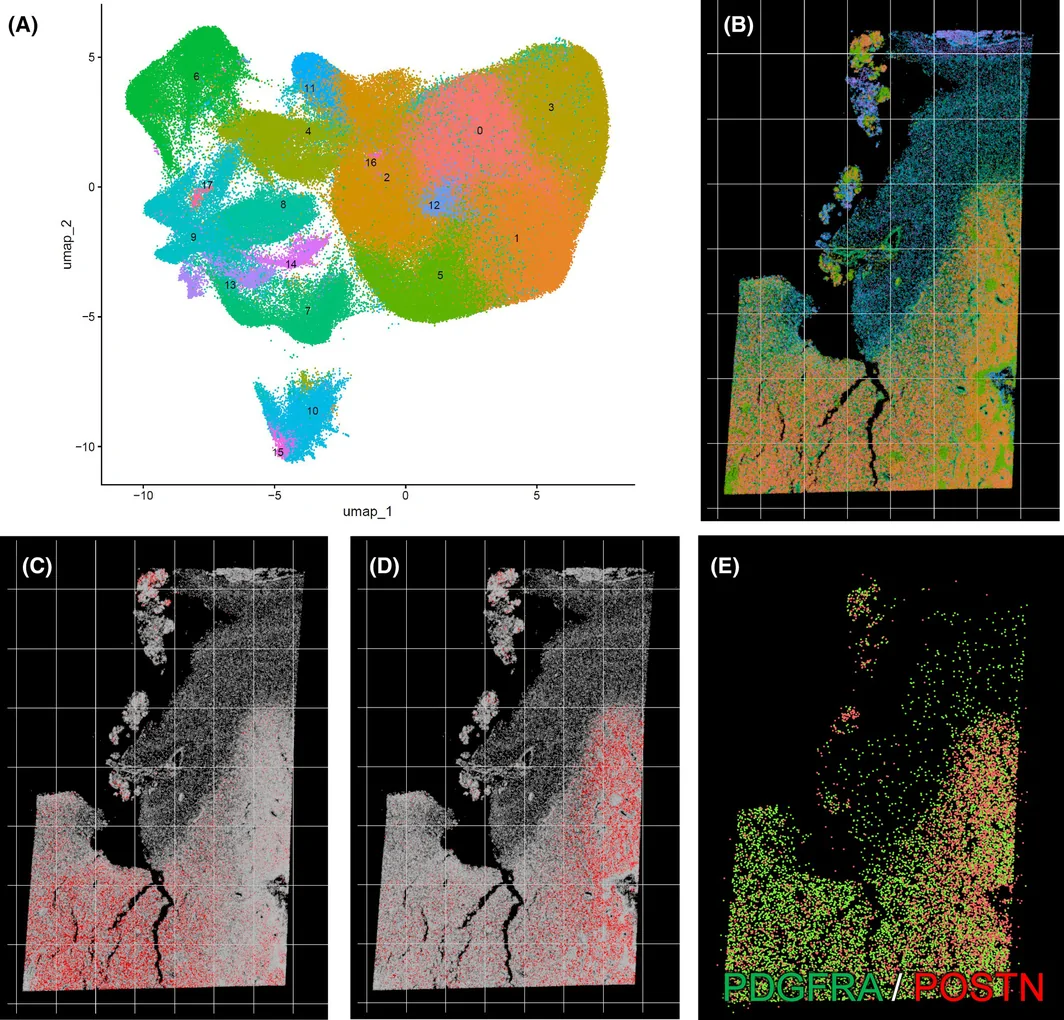
Spatial transcriptomic characterization of glioblastoma, IDH‐wildtype. UMAP visualization demonstrating unsupervised clustering of Xenium transcriptomic data into 18 clusters, including heterogeneous tumor cell populations (Clusters 0–3, 5, and 12), neuronal populations (Clusters 9, 10, 14, and 15), reactive astrocytes (Cluster 6), oligodendrocytes (Cluster 8), endothelial cells (Cluster 7), and immune‐associated cells (Cluster 4) (A). Several clusters could not be classified confidently because of the limited number of predefined genes in this study. Spatial mapping of the identified clusters within the tissue section exhibited preserved histological architecture and marked spatial intratumoral heterogeneity (B). Spatial mapping of tumor cell clusters 0 (C) and 1 (D) highlights spatial intratumoral transcriptional heterogeneity. Spatial expression pattern of *POSTN* (red dots) demonstrated preferential expression in *PDGFRA*‐positive tumor cell populations (green dots), corresponding to cluster 1 (E).

*TGFB2*‐associated TGFβ/BMP signaling pathways were enriched predominantly in Cluster 0 (Tables S1 and S2), although *TGFB2* transcripts were detected across multiple tumor‐associated clusters (Figure S4). We therefore examined whether neurons spatially associated with *TGFB2*‐enriched Cluster 0 tumor cells showed localized neuronal transcriptional alterations and TGFβ‐related signaling.

Representative infiltrative and remote cortical regions are shown in Figure 2A. Histological evaluation identified morphologically viable residual neurons entrapped within infiltrative tumor tissue (Figure 2B), whereas neurons in the remote cortex showed preserved architecture (Figure 2C). During exploratory spatial transcriptomic analysis, *IGFBP5* was identified as preferentially expressed in tumor‐associated neuronal populations and was selected for further evaluation. IGFBP5 expression was mainly observed in *SLC17A7*‐positive neuronal populations within glioma‐infiltrated regions (Figure 2D). Among the neuronal markers examined, *SLC17A7* showed the clearest correspondence with morphologically identifiable entrapped neurons. Combined visualization showed close spatial juxtaposition between *SLC17A7*‐positive/IGFBP5‐positive neurons and adjacent *PDGFRA*‐positive tumor cells (Figure 2D). In contrast, neurons in remote non‐infiltrated regions showed substantially lower *IGFBP5* expression (Figure 2E). Quantitative comparison confirmed significantly higher *IGFBP5* expression among *SLC17A7*‐positive neuronal populations in the analyzed infiltrated region than in the selected remote cortical region (Figure S5).

**FIGURE 2:**
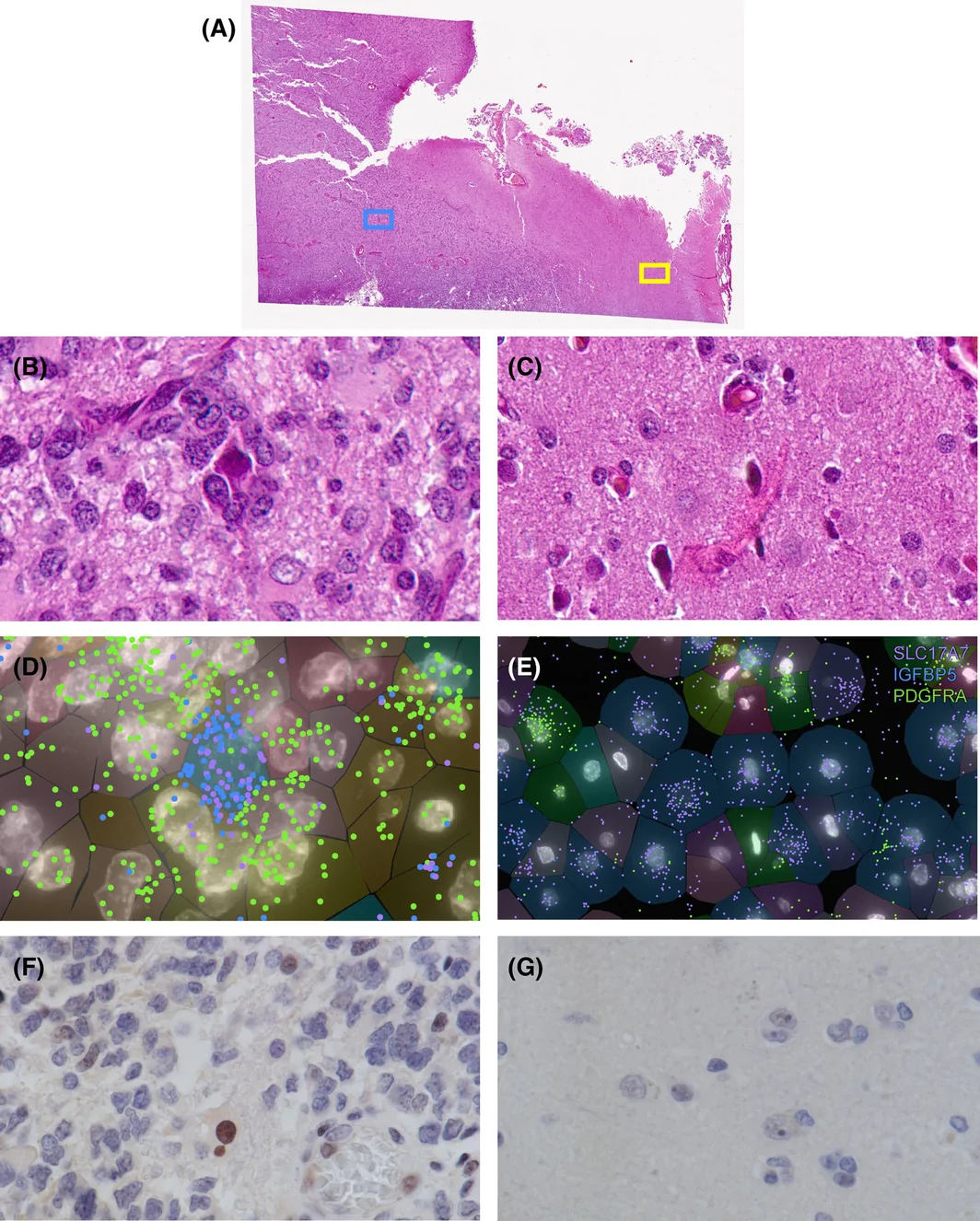
Spatial transcriptomic and immunohistochemical visualization of localized neuronal state alterations in glioma‐infiltrated regions. Low‐magnification hematoxylin and eosin staining images illustrating the infiltrative glioma architecture. Blue rectangles indicate glioma‐infiltrated regions at the infiltrative tumor front, where tumor cells surround and entrap residual neuronal populations, corresponding to panels B, D, and F. Yellow rectangles indicate remote non‐infiltrated cerebral cortical regions distant from the tumor, corresponding to panels C, E, and G (A). Higher magnification hematoxylin and eosin images of glioma‐infiltrated tissue demonstrating morphologically viable residual neurons entrapped within the infiltrating tumor cells (B). The corresponding remote non‐infiltrated cerebral cortex showing preserved neuronal architecture (C). Among the neuronal markers included in the Xenium panel, *SLC17A7* showed the clearest spatial correspondence with morphologically identifiable entrapped neuronal populations within infiltrative tumor regions (D). In contrast, neurons in the remote non‐infiltrated regions lacked comparable *IGFBP5* expression (E). Immunohistochemical analysis identified pSMAD2‐positive neuronal populations adjacent to infiltrating tumor cells within glioma‐infiltrated regions (F), whereas neurons located in remote non‐infiltrated regions lacked detectable pSMAD2 expression (G).

*PDGFRA*‐positive tumor cells infiltrated around these residual neurons, resulting in close spatial juxtaposition between tumor cells and *IGFBP5*‐positive neuronal populations. Notably, these neuronal populations were preferentially localized within tumor regions corresponding to Cluster 0, which demonstrated enrichment of *TGFB2*‐associated TGFβ/BMP signaling pathways. Proliferative activity appeared comparable among adjacent tumor cells.

We next examined whether neurons within glioma‐infiltrated regions showed localized TGFβ‐related signaling. Immunohistochemical analysis identified pSMAD2‐positive neuronal populations adjacent to infiltrating tumor cells (Figure 2F), whereas neurons in remote non‐infiltrated regions lacked detectable pSMAD2 expression (Figure 2G). In serial sections, pSMAD2‐positive neurons and *IGFBP5*‐expressing *SLC17A7*‐positive neurons were observed in closely corresponding fields within the same glioma‐infiltrated regions. Because immunohistochemistry and Xenium analysis were performed on different sections, direct single‐cell‐level colocalization could not be confirmed. Nevertheless, their close spatial correspondence supports the interpretation that *IGFBP5* expression and TGFβ‐related signaling occurred within the same tumor‐associated neuronal population. These findings suggest localized neuronal transcriptional state alterations associated with the glioma microenvironment. Four additional high‐grade diffuse glioma cases were examined for pSMAD2 expression. Two showed significantly higher proportions of pSMAD2‐positive neurons in the glioma‐infiltrated regions, whereas the other two did not (Figure S6), indicating that localized neuronal SMAD2 phosphorylation occurred in a subset of cases.

The present findings suggest that TGFβ‐related signaling may be active in tumor‐associated neurons within glioma‐infiltrated regions, as supported by pSMAD2‐positive neuronal populations adjacent to infiltrating tumor cells. Notably, these neurons represented morphologically viable residual neurons embedded within infiltrative tumor tissue, rather than degenerating neurons associated with necrotic regions. Thus, localized neuronal transcriptional alterations may occur in viable neurons exposed to the glioma microenvironment.

*IGFBP5* has been implicated in extracellular matrix remodeling and fibrosis‐associated transcriptional responses in multiple tissues and tumors [6]. Similarly, TGFβ signaling is a major regulator of extracellular matrix‐associated and mesenchymal‐like transcriptional programs and has been associated with mesenchymal states in gliomas [7]. Although interactions between TGFβ‐related pathways and *IGFBP5*‐associated biological processes have been suggested in fibrosis‐associated conditions [8], the spatial association between TGFβ‐related signaling and neuronal IGFBP5 expression within infiltrative glioma tissue has not, to our knowledge, been previously described. The biological significance of these observations remains unclear and requires further investigation.

This study had several limitations. First, Xenium‐based spatial transcriptomics demonstrates spatially associated transcriptional states within preserved tissue architecture but does not establish definitive molecular interactions or causal biological relationships. Second, the predefined and relatively restricted gene panel limited comprehensive ligand–receptor‐based cell–cell interaction analysis. Additionally, differences in spatial resolution among platforms may limit direct visualization of the cell‐level relationships observed here. Therefore, the observed spatial association among *TGFB2*‐expressing tumor cells, pSMAD2‐positive neurons, and *IGFBP5*‐expressing neurons should be interpreted as hypothesis‐generating rather than as definitive evidence of a specific signaling pathway. Third, the restricted gene panel did not permit comprehensive characterization of neuronal subtypes across brain regions. SLC17A7 was selected because it showed the clearest correspondence with morphologically identifiable residual neurons. Complementary immunohistochemical analysis of phosphorylated SMAD2 provided orthogonal evidence supporting localized TGFβ‐related signaling activity in tumor‐associated neurons.

Collectively, neurons within glioma‐infiltrated tissue showed localized transcriptional alterations accompanied by evidence of TGFβ‐related signaling, highlighting the utility of Xenium‐based spatial transcriptomics for visualizing tumor‐associated neuronal states within preserved infiltrative glioma architecture. Integrating spatial transcriptomics with morphology‐ and protein‐based pathological analyses may provide a valuable hypothesis‐generating framework for investigating localized tumor microenvironmental interactions in human gliomas.

## AUTHOR CONTRIBUTIONS

Kaishi Satomi and Junji Shibahara contributed to the pathological diagnosis. Kaishi Satomi and Hiromi Onizuka generated, analyzed, and interpreted the spatial transcriptomic and molecular data. Kaishi Satomi, Yutaka Suzuki, and Koichi Ichimura performed spatial transcriptome analyses with support from Hirokazu Takami and Takako Yoshioka. Kuniaki Saito and Motoo Nagane provided clinical samples and clinical data. Kaishi Satomi, Hirokazu Takami, Koichi Ichimura, and Junji Shibahara drafted the manuscript with contributions from all the authors. All authors have reviewed and approved the final manuscript.

## CONFLICT OF INTEREST STATEMENT

The authors declare no conflicts of interest.

## Supporting information


**Figure S1.** DNA methylation‐based classification and copy number analysis of the present case. DNA methylation profiling using the Epignostix CNS Tumor Classifier version 12.8.1 yielded a calibrated score of 0.88478 for “Pediatric‐type diffuse high‐grade gliomas,” without assignment to a specific methylation class (A). Copy number analysis demonstrated *PDGFRA* amplification together with homozygous deletion of *CDKN2A* and *CDKN2B* (B).
**FIGURE S2.** UMAP visualization of representative gene expression patterns. *PDGFRA* expression was broadly observed across tumor cell populations (A). *SLC17A7* expression was predominantly observed in neuronal populations (B). *IGFBP5* expression was observed across multiple clusters, with enrichment in selected neuronal (Cluster 9) and neoplastic cell populations (C). *VIM* expression was widely distributed across tumor‐associated cellular populations (D). *POSTN* expression demonstrated preferential enrichment in a distinct tumor‐associated subpopulation (E). In contrast, *TGFB2* expression showed a largely mutually exclusive distribution pattern (F).
**FIGURE S3.** Correlation between histological architecture and *POSTN* expression with Xenium‐based cell segmentation. Low‐power hematoxylin and eosin image of the analyzed tissue section (A) and corresponding *POSTN* expression map (red) (B). Higher magnification view of the tumor region (C) and corresponding *POSTN* expression with preferential localization in relatively deep tumor regions, including areas containing vascular structures (D).
**FIGURE S4.** Violin plots showing expression of representative genes across the identified clusters. Expression distributions of *PDGFRA* (A), *TGFB2* (B), *POSTN* (C), and *IGFBP5* (D) across clusters identified by unsupervised clustering. *PDGFRA* was broadly expressed across tumor‐associated clusters. *TGFB2* expression was higher in Cluster 0 than Cluster 1, whereas *POSTN* expression was preferentially enriched in Cluster 1.
**FIGURE S5.** Quantitative comparison of *IGFBP5* expression in *SLC17A7*‐positive neuronal populations. Spatial transcriptomic overview of *SLC17A7*‐positive neuronal populations (magenta) and *IGFBP5* expression (blue) showing the locations of glioma‐infiltrated and remote cortical regions selected for analysis (A). Representative high‐magnification views demonstrating co‐localization of *SLC17A7* and *IGFBP5* within remote non‐infiltrated cortex (B) and glioma‐infiltrated regions (C). Violin plot showing *IGFBP5* expression levels among *SLC17A7*‐positive neuronal populations from the selected regions (D). Within the analyzed regions, *IGFBP5* expression was higher in *SLC17A7*‐positive neurons from glioma‐infiltrated tissue than in those from remote cortex (Wilcoxon rank‐sum test, *P* = 7.99 × 10^−12^).
**FIGURE S6.** The proportions of pSMAD2‐positive neurons were compared between remote cortical regions (R) and glioma‐infiltrated regions (G) in the present case and four additional cases. Ten microscopic fields were evaluated for each region in each case. Each dot represents an individual microscopic field, and horizontal bars indicate medians. *P* values were calculated using two‐sided Mann–Whitney *U* tests. *P < 0.05; **P < 0.01; n.s., not significant.

Supporting Information.

## Data Availability

The data that support the findings of this study are available from the corresponding author upon reasonable request.
