# Supplementary material for: Spatial transcriptomic analysis of neurons at the glioma infiltration front: A case study
Source: Brain Pathol. 2026 Sep 29:e70154. Online ahead of print. doi: 10.1111/bpa.70154 (PMC13621363; doi:10.1111/bpa.70154)
Supplement: Supplementary file 1 [file BPA-9999-e70154-s002.pdf]

SUPPLEMENTARY FIGURE 1

A

Predictions	Calibrated score	Interpretation	
Paediatric-type diffuse high-grade gliomas	0.88478	no match	✗
Diffuse pediatric-type high-grade glioma, H3-wildtype and IDH-wildtype	0.8745	no match	✗
Diffuse paediatric-type high grade glioma, RTK1 subtype	0.87326	no match	✗
MC Diffuse paediatric-type high grade glioma, RTK1 subtype, subclass C (novel)	0.84931	no match	✗

B

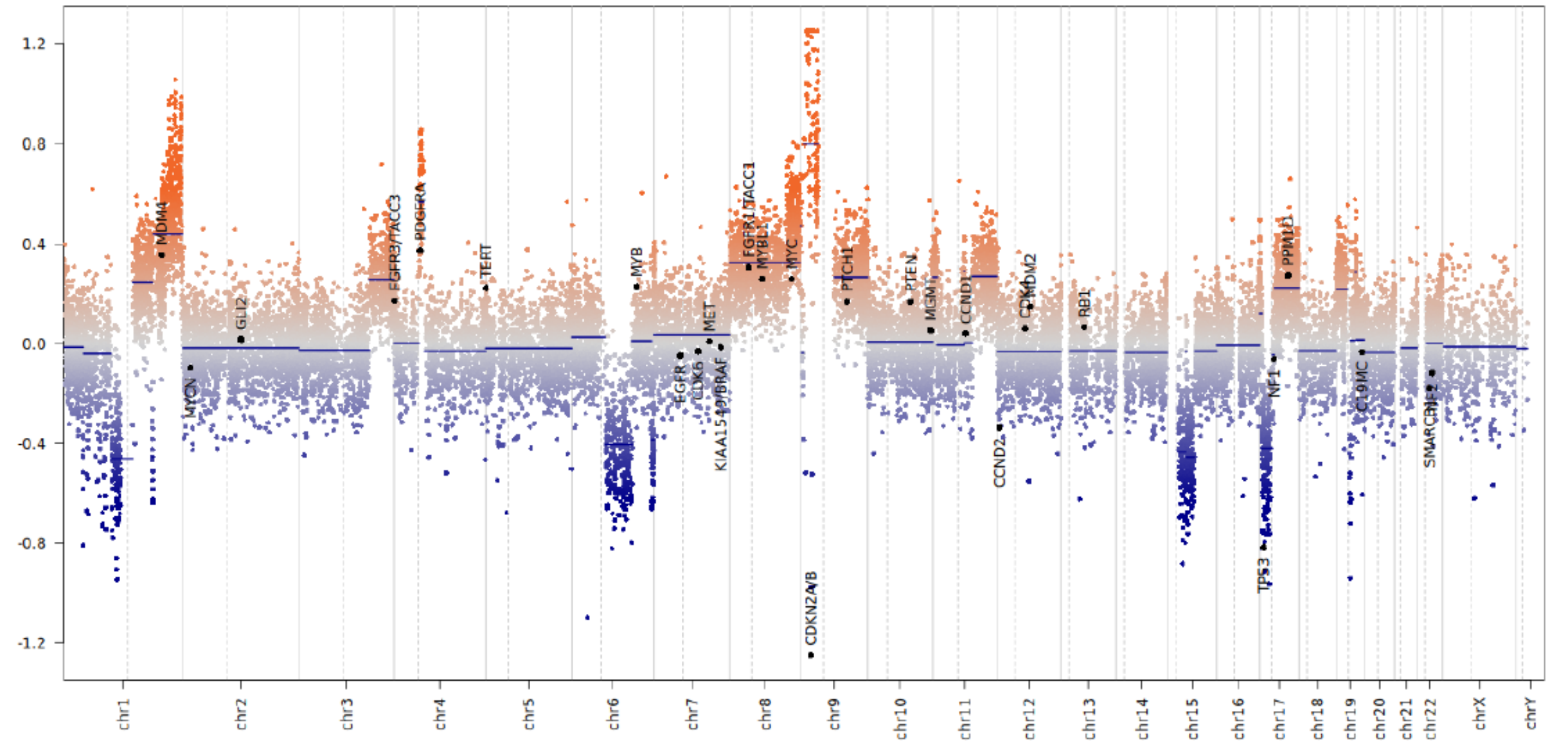

SUPPLEMENTARY FIGURE 1. DNA methylation-based classification and copy number analysis of the present case.
DNA methylation profiling using the Epignostix CNS Tumor Classifier version 12.8.1 yielded a calibrated score of 0.88478 for “Pediatric-type diffuse high-grade gliomas”, without assignment to a specific methylation class (A). Copy number analysis demonstrated *PDGFRA* amplification together with homozygous deletion of *CDKN2A* and *CDKN2B* (B).

SUPPLEMENTARY FIGURE 2

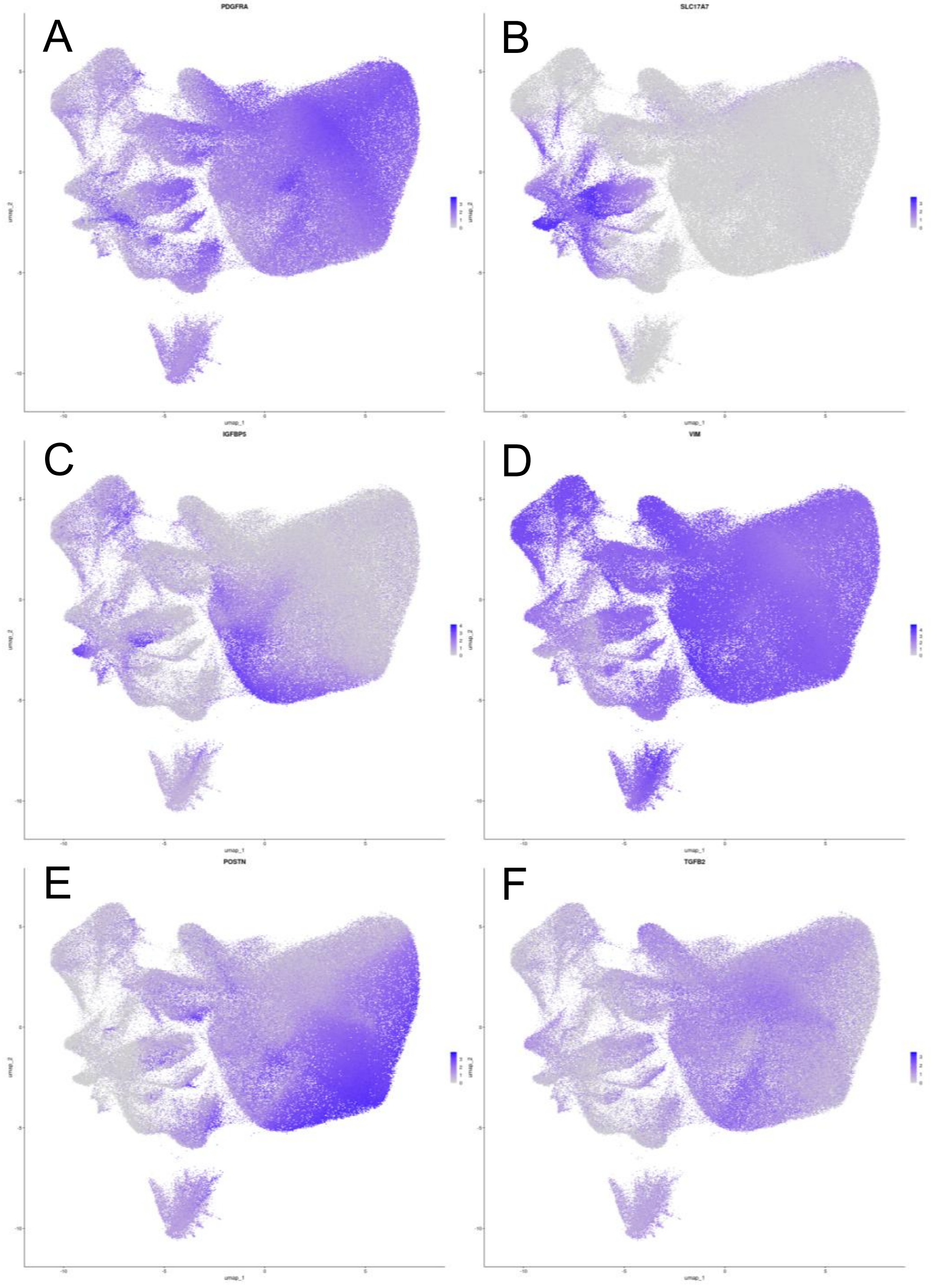

SUPPLEMENTARY FIGURE 2. UMAP visualization of representative gene expression patterns.

PDGFRA expression was broadly observed across tumor cell populations (A). *SLC17A7* expression was predominantly observed in neuronal populations (B). *IGFBP5* expression was observed across multiple clusters, with enrichment in selected neuronal (Cluster 9) and neoplastic cell populations (C). *VIM* expression was widely distributed across tumor-associated cellular populations (D). *POSTN* expression demonstrated preferential enrichment in a distinct tumor-associated subpopulation (E). In contrast, *TGFB2* expression showed a largely mutually exclusive distribution pattern (F).

SUPPLEMENTARY FIGURE 3

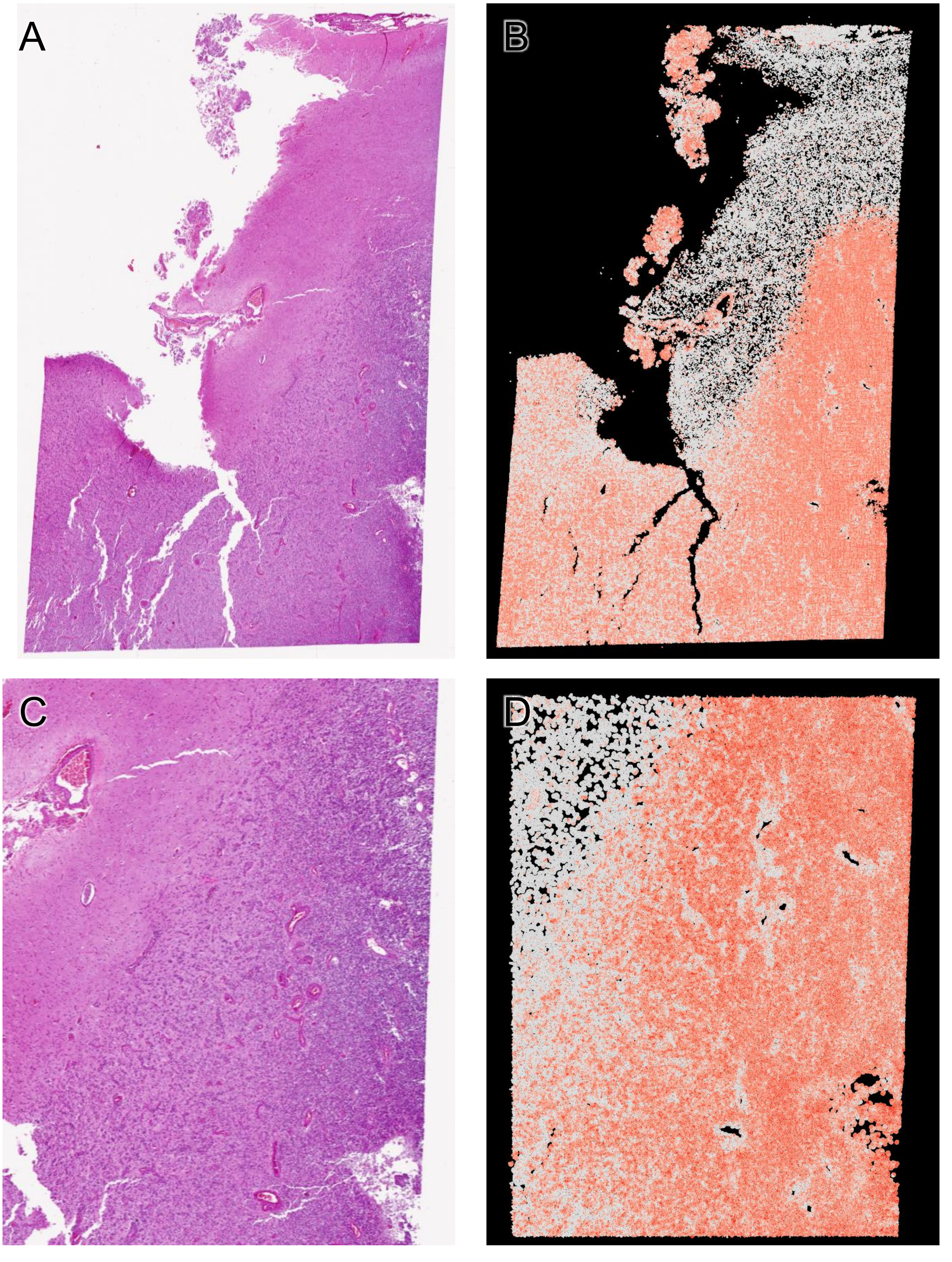

SUPPLEMENTARY FIGURE 3 Correlation between histological architecture and *POSTN* expression with Xenium-based cell segmentation. Low-power hematoxylin and eosin image of the analyzed tissue section (A) and corresponding *POSTN* expression map (red) (B). Higher magnification view of the tumor region (C) and corresponding *POSTN* expression with preferential localization in relatively deep tumor regions, including areas containing vascular structures (D).

SUPPLEMENTARY FIGURE 4

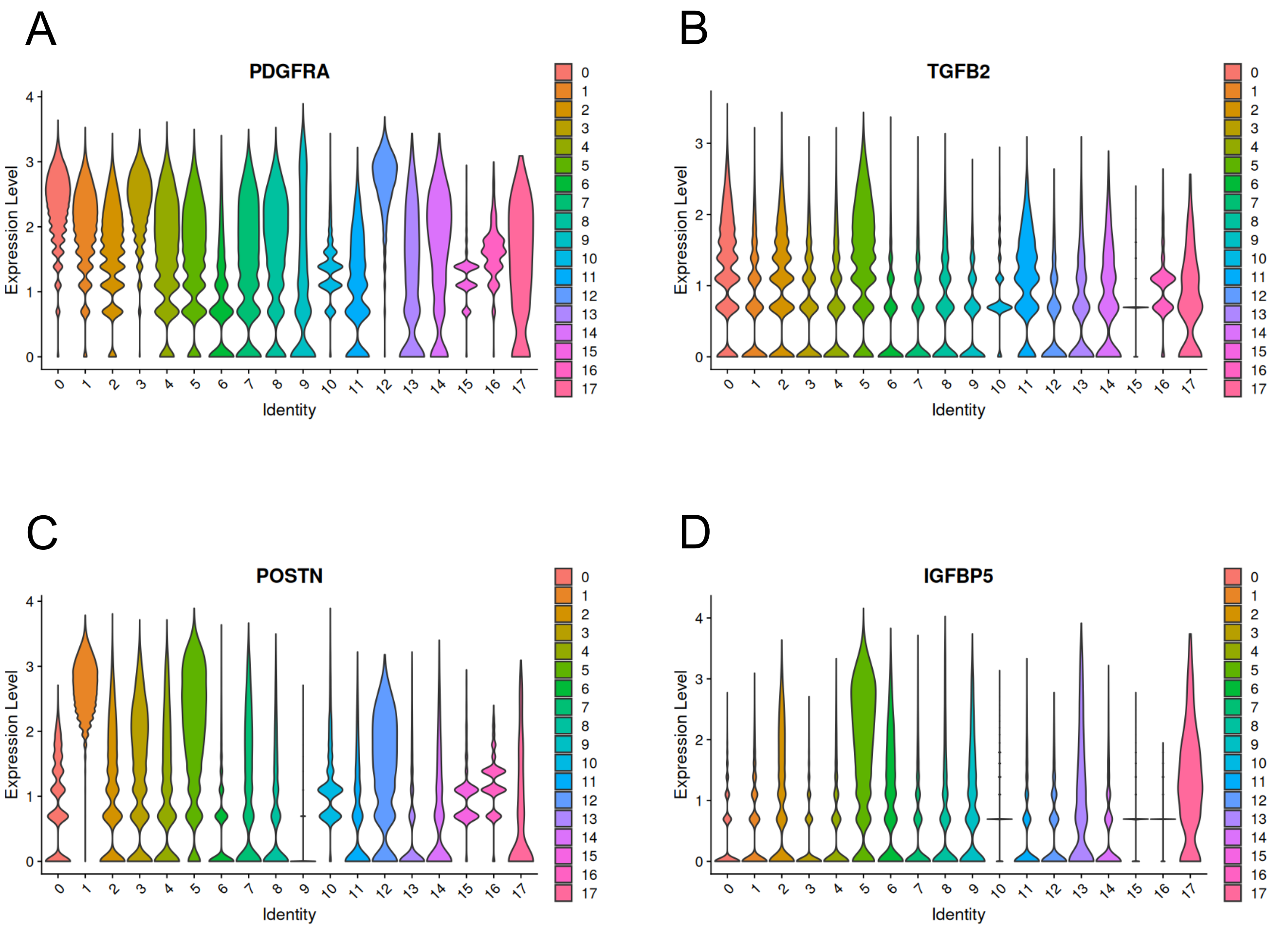

SUPPLEMENTARY FIGURE 4. Violin plots showing expression of representative genes across the identified clusters.
Expression distributions of *PDGFRA* (A), *TGFB2* (B), *POSTN* (C), and *IGFBP5* (D) across clusters identified by unsupervised clustering. *PDGFRA* was broadly expressed across tumor-associated clusters. *TGFB2* expression was higher in Cluster 0 than Cluster 1, whereas *POSTN* expression was preferentially enriched in Cluster 1.

SUPPLEMENTARY FIGURE 5

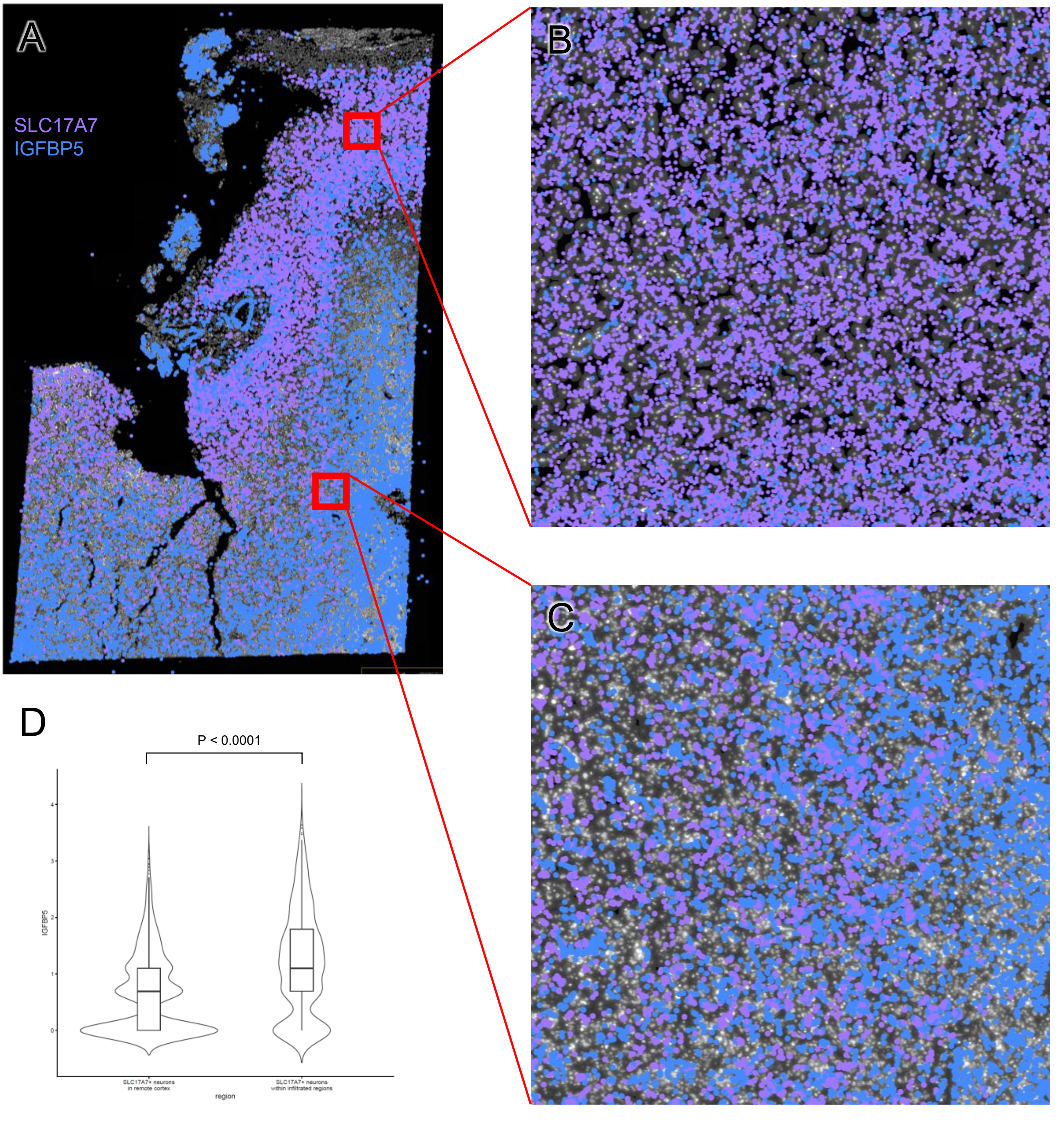

SUPPLEMENTARY FIGURE 5 Quantitative comparison of *IGFBP5* expression in *SLC17A7*-positive neuronal populations.
Spatial transcriptomic overview of *SLC17A7*-positive neuronal populations (magenta) and IGFBP5 expression (blue) showing the locations of glioma-infiltrated and remote cortical regions selected for analysis (A). Representative high-magnification views demonstrating co-localization of *SLC17A7* and *IGFBP5* within remote non-infiltrated cortex (B) and glioma-infiltrated regions (C). Violin plot showing *IGFBP5* expression levels among *SLC17A7*-positive neuronal populations from the selected regions (D). Within the analyzed regions, *IGFBP5* expression was higher in *SLC17A7*-positive neurons from glioma-infiltrated tissue than in those from remote cortex (Wilcoxon rank-sum test, $P = 7.99 \times 10^{-12}$).

SUPPLEMENTARY FIGURE 6

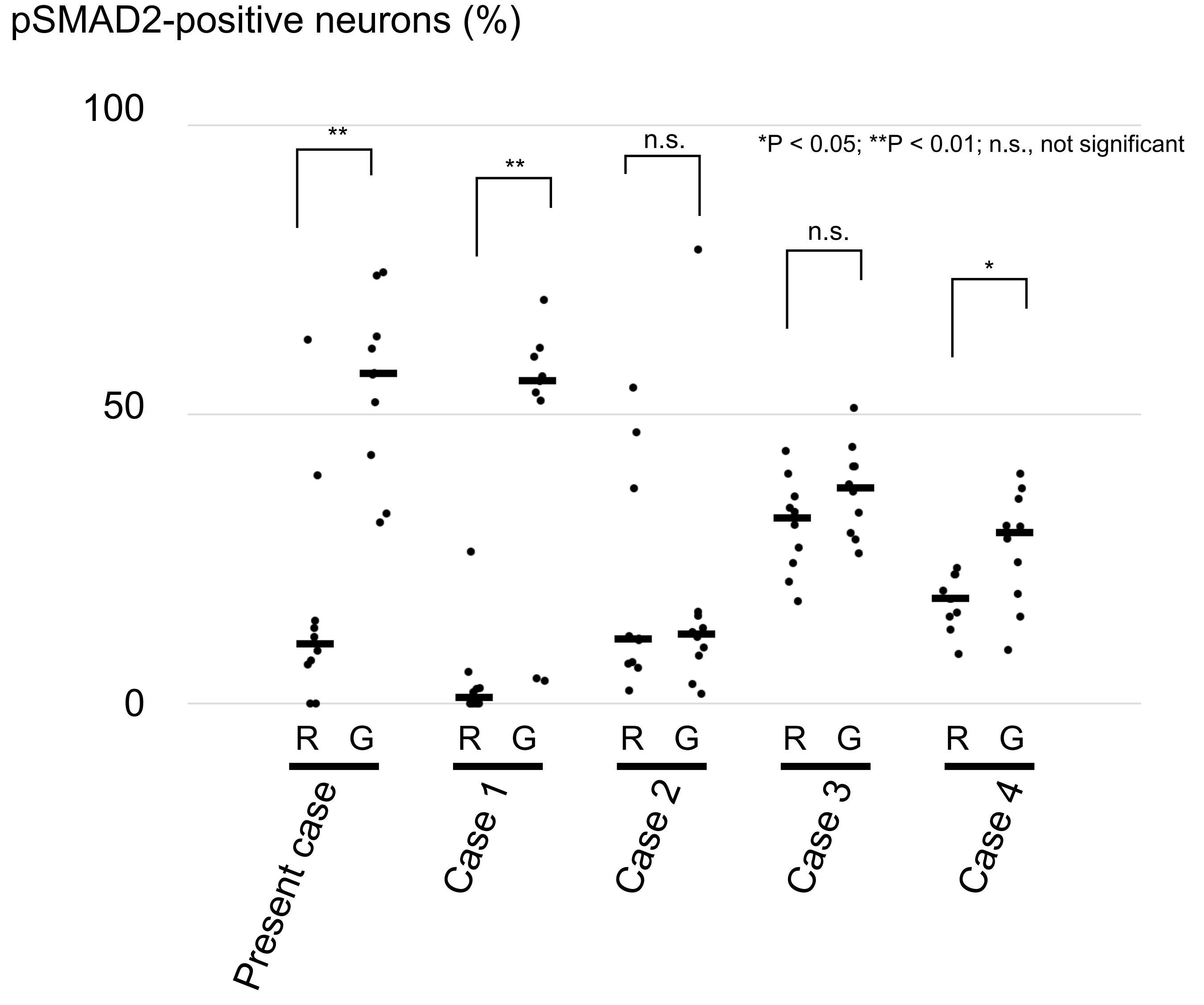

SUPPLEMENTARY FIGURE 6 The proportions of pSMAD2-positive neurons were compared between remote cortical regions (R) and glioma-infiltrated regions (G) in the present case and four additional cases. Ten microscopic fields were evaluated for each region in each case. Each dot represents an individual microscopic field, and horizontal bars indicate medians. P values were calculated using two-sided Mann–Whitney U tests. *P < 0.05; **P < 0.01; n.s., not significant.
